# Impact of Structured Physical Exercise Programs (SPEs) on Symptoms and Clinical Outcomes in Schizophrenia: A Systematic Review With Qualitative Synthesis of Randomized Controlled Trials (2015–2025)

**DOI:** 10.62641/aep.v54i3.2198

**Published:** 2026-06-15

**Authors:** Gustavo Baroni Araujo, Carolina Alves de Moraes Nicolau, Bruno Marson Maladogi, Sionaldo Eduardo Ferreira, Christian José dos Santos, Helio Serassuelo Junior

**Affiliations:** ^1^Associated Postgraduate Program in Physical Education, State University of Londrina (UEL), 86057-970 Londrina, Paraná, Brazil; ^2^Department of Sports Sciences, State University of Londrina (UEL), 86057-970 Londrina, Paraná, Brazil; ^3^Department of Biochemistry, Pharmacology and Physiology, Federal University of Triângulo Mineiro (UFTM), 38025-440 Uberaba, Minas Gerais, Brazil; ^4^Postgraduate Program in Biological Sciences, Federal University of Goiás (UFG), 74690-900 Goiânia, Goiás, Brazil

**Keywords:** schizophrenia, exercise, physical fitness, cognition, qualitative synthesis, randomized controlled trials

## Abstract

**Background:**

People with schizophrenia present persistent deficits that are poorly responsive to pharmacological treatment, particularly including negative symptoms and cognition. Recent evidence indicates that structured physical exercise programs (SPEs) can modulate relevant neurobiological mechanisms and improve essential clinical outcomes. Given the heterogeneity of existing protocols, a systematic synthesis of this evidence is necessary. The aim of this study was to investigate the effects of SPEs on symptomatology, cognition, global functioning, and physical fitness in individuals with schizophrenia.

**Methods:**

A systematic review was performed according to the Preferred Reporting Items for Systematic Reviews and Meta-Analyses (PRISMA) guidelines, with searches conducted in the PubMed/Medline, Scopus, Web of Science, Cochrane Central Register of Controlled Trials (CENTRAL), Scientific Electronic Library Online (SciELO), Latin American and Caribbean Health Sciences Literature (LILACS), and Virtual Health Library (VHL) databases. Randomized clinical trials with adults diagnosed with schizophrenia who underwent structured exercise interventions were included. Initial searches identified 756 records, of which 16 studies were included in the review and comprised the final synthesis. Data extraction followed a standardized form, and study characteristics are described using descriptive statistics.

**Results:**

The included trials demonstrated consistent effects of different categories of physical exercise on negative symptoms, general symptoms, and overall functioning related to physical fitness, as well as improvements in cognitive aspects. A variety of SPE protocols were observed, as well as different intensities and instruments for evaluating the effectiveness of the interventions. Regarding intensity, it was noted that low-intensity SPEs also produced benefits, especially in institutionalized contexts; however, the metabolic effects were less consistent. Furthermore, the heterogeneity of the protocols influenced the magnitude of the results.

**Conclusions:**

SPEs generate positive clinical impacts that are adaptable to the needs of each patient, an essential aspect given the common barriers observed in the diagnosis of schizophrenia. The findings support the integration of SPEs into mental health services, as a component capable of expanding clinical and functional outcomes, and offering an accessible intervention aligned with the needs of this population.

## Introduction

Schizophrenia remains one of the most disabling psychiatric disorders, not only because of the presence of positive psychotic symptoms but also due to the persistence of negative symptoms and cognitive deficits that permanently compromise the social and occupational functioning and autonomy of affected individuals [1]. Robust evidence demonstrates that these symptomatic domains are more strongly associated with functional outcomes and quality of life than the positive symptoms themselves, thus constituting priority therapeutic targets that have historically been neglected in clinical practice [2].

Although antipsychotic medications represent the cornerstone of schizophrenia treatment, their effectiveness presents well-recognized limitations. Meta-analyses indicate that these drugs primarily reduce positive symptoms, while their effects on negative symptoms and cognitive deficits are generally modest and often insufficient to produce meaningful functional recovery [3, 4]. In addition, long-term pharmacological treatment is frequently associated with metabolic and cardiovascular side effects that contribute to reduced physical fitness and an increased risk of morbidity and mortality in this population [5]. These limitations highlight the importance of complementary non-pharmacological strategies that are able to address broader clinical and functional dimensions of the disorder.

For the purposes of the current review, structured physical exercise programs (SPEs) are defined as planned, structured, repetitive, and purposeful physical activities, aimed at improving or maintaining physical fitness components, such as cardiorespiratory capacity, muscular strength, and endurance. This definition is consistent with widely adopted conceptual frameworks in exercise science and public health literature, which distinguish physical exercise from general physical activity based on its systematic and goal-oriented nature [6]. Physical exercise promotes widely documented physiological and neurobiological adaptations, including increased cardiorespiratory fitness, modulation of dopaminergic and glutamatergic systems, enhancement of neuroplasticity, and reduction in systemic inflammatory processes, mechanisms closely related to the pathophysiology of schizophrenia [7].

Over the past decade, several randomized clinical trials have investigated the effects of different exercise modalities on psychiatric symptoms, cognitive functioning, physical fitness, and other clinically relevant outcomes in individuals with schizophrenia. Previous systematic reviews and meta-analyses suggest that exercise-based interventions may improve negative symptoms, cognitive performance, and global functioning, in addition to producing benefits for physical health indicators [8, 9, 10]. However, considerable heterogeneity exists among intervention protocols, including differences in exercise modality, intensity, duration, supervision, and outcome measures, which makes direct comparisons between studies challenging.

Furthermore, many previous reviews combine evidence from studies with heterogeneous designs, including observational studies and uncontrolled interventions, which may limit their ability to establish robust causal inferences regarding the therapeutic effects of exercise in schizophrenia [11, 12]. In this sense, analyses that focus exclusively on randomized clinical trials are essential to provide a more rigorous synthesis of the available evidence on the clinical effects of structured exercise interventions.

Given this scenario, there is a need to critically organize the available evidence derived from randomized clinical trials, in order to better understand the extent to which physical exercise interventions influence psychiatric symptoms, cognition, physical fitness, and other clinical indicators of schizophrenia. Such an approach allows not only evaluation of the magnitude and consistency of reported effects but also identification of methodological gaps and directions for future research.

Therefore, the aim of the current study was to systematically synthesize the evidence from randomized clinical trials that investigated structured physical exercise programs in people with schizophrenia, to evaluate their effects on psychiatric symptoms and other relevant clinical outcomes, as well as identify response patterns associated with different exercise modalities and intervention protocols.

## Materials and Methods

### Study Design

This systematic review was conducted in accordance with the Preferred Reporting Items for Systematic Reviews and Meta-Analyses (PRISMA 2020) guidelines [12], ensuring methodological rigor and transparency throughout all stages of the review.

### Research Question and Eligibility Criteria

The research question was structured according to the Population, Intervention, Comparison, and Outcomes (PICO) model. The Population (P) included adults (≥18 years) diagnosed with schizophrenia based on Diagnostic and Statistical Manual of Mental Disorders (DSM) or International Classification of Diseases (ICD) criteria, or explicit clinical diagnostic descriptions. Studies including mixed-age populations were only considered if data for adult participants were reported separately. The Intervention (I) consisted of structured physical exercise programs, supervised or unsupervised, including aerobic training, resistance training, high-intensity interval training (HIIT), combined exercise protocols, or other structured physical activity interventions described in the trials. The Comparison (C) included usual care, waiting list conditions, non-exercise interventions, or comparisons between different exercise modalities and intensities. Outcomes (O) comprised changes in psychiatric symptoms (positive, negative, and general symptoms) as well as additional clinical outcomes, such as cognition, physical fitness, functioning, and biological markers, assessed through validated instruments. The Study type (S) was restricted to randomized controlled trials (RCTs).

Eligibility criteria were defined a priori to ensure methodological transparency and reduce potential selection bias. Studies were included if they met the following criteria: (1) RCTs published in peer-reviewed journals; (2) studies involving adults (≥18 years) diagnosed with schizophrenia or schizophrenia spectrum disorders, or studies with mixed-age samples, provided that results for adult participants were reported separately; (3) investigations evaluating structured physical exercise interventions with clearly described protocols, including information on frequency, duration, intensity, or modality of exercise; and (4) studies reporting at least one clinical outcome related to psychiatric symptoms, cognitive performance, physical fitness, global functioning, or other relevant health indicators.

Studies were excluded if they met any of the following criteria: (1) observational studies, quasi-experimental designs, case reports, reviews, study protocols, theses, or dissertations; (2) studies involving mixed psychiatric populations without separate analysis for participants with schizophrenia; (3) studies evaluating general physical activity or lifestyle interventions without a structured exercise protocol; or (4) studies that did not report post-intervention clinical outcomes.

### Search Strategy

The systematic search was conducted in the PubMed/Medline, Scopus, Web of Science, Cochrane Central Register of Controlled Trials (CENTRAL), Scientific Electronic Library Online (SciELO), Latin American and Caribbean Health Sciences Literature (LILACS), and Virtual Health Library (VHL) databases. These databases were selected due to their broad coverage of psychiatry, neuroscience, and exercise science literature.

The search period covered studies published between January 2015 and September 2025 in order to capture recent and methodologically relevant evidence. Controlled descriptors Medical Subject Headings/Health Sciences Descriptors (MeSH/DeCS) and free-text terms related to “schizophrenia,” “exercise,” “physical activity,” and “randomized controlled trial” were combined using Boolean operators (AND/OR). Database-specific adaptations were applied when necessary. When available, filters for human participants, adults, and RCTs were used.

### Study Selection

All records retrieved from the databases were imported into the Zotero reference manager, where duplicate records were removed using automated detection followed by manual verification. Study selection was conducted in two stages; first, titles and abstracts were screened independently by two reviewers, then second, the full texts of potentially eligible studies were assessed. Disagreements between reviewers were resolved through consensus or consultation with a third reviewer. Reasons for exclusion during the full-text stage were recorded.

### Data Extraction

Data extraction was performed independently by two reviewers using a standardized data extraction form. The information collected included author and year, country, study design, sample characteristics, diagnostic criteria, clinical setting (outpatient, community, or inpatient), characteristics of the intervention (exercise modality, frequency, duration, and intensity when reported), type of comparator group, instruments used to assess outcomes, and main clinical outcomes. When available, additional information regarding adherence rates, participant dropouts, and adverse events was also extracted.

### Risk of Bias Assessment

The risk of bias of the included studies was assessed using the Cochrane Risk of Bias 2.0 (RoB 2) tool [13, 14]. This instrument assesses domains related to the randomization process, deviations from intended interventions, missing outcome data, outcome measurements, and selection of reported results. The risk of bias assessments were incorporated into the critical interpretation of the findings.

### Data Synthesis

Due to the considerable heterogeneity among the included studies in terms of exercise modality (e.g., aerobic training, resistance training, HIIT, Tai Chi, and combined exercise programs), intervention intensity, duration, clinical settings (outpatient versus institutionalized populations), and outcome assessment instruments, a quantitative meta-analysis was not considered methodologically appropriate. In addition, the trials evaluated different outcome domains using heterogeneous measurement tools (e.g., Positive and Negative Syndrome Scale (PANSS), Brief Psychiatric Rating Scale (BPRS), MATRICS Consensus Cognitive Battery (MCCB), maximal oxygen consumption (VO_2_ max), functional scales, and biological markers), which limited the possibility of calculating and pooling comparable effect sizes across studies. Conducting a meta-analysis under such conditions could lead to misleading statistical aggregation and compromise the interpretability of the results.

Therefore, the synthesis of results was conducted qualitatively. The findings were organized according to major outcome domains, including positive symptoms, negative symptoms, general psychopathology, cognitive outcomes, functional outcomes, and physiological variables.

To improve interpretability of comparisons, control conditions were categorized into three types: (1) usual care or treatment as usual (TAU); (2) passive controls, such as waiting list conditions; and (3) active non-exercise interventions (e.g., psychoeducation, cognitive training, or active video games). This stratification allows clearer interpretations of intervention effects, since comparisons with passive controls tend to produce larger effect sizes than those involving active interventions.

**Fig. 1.**
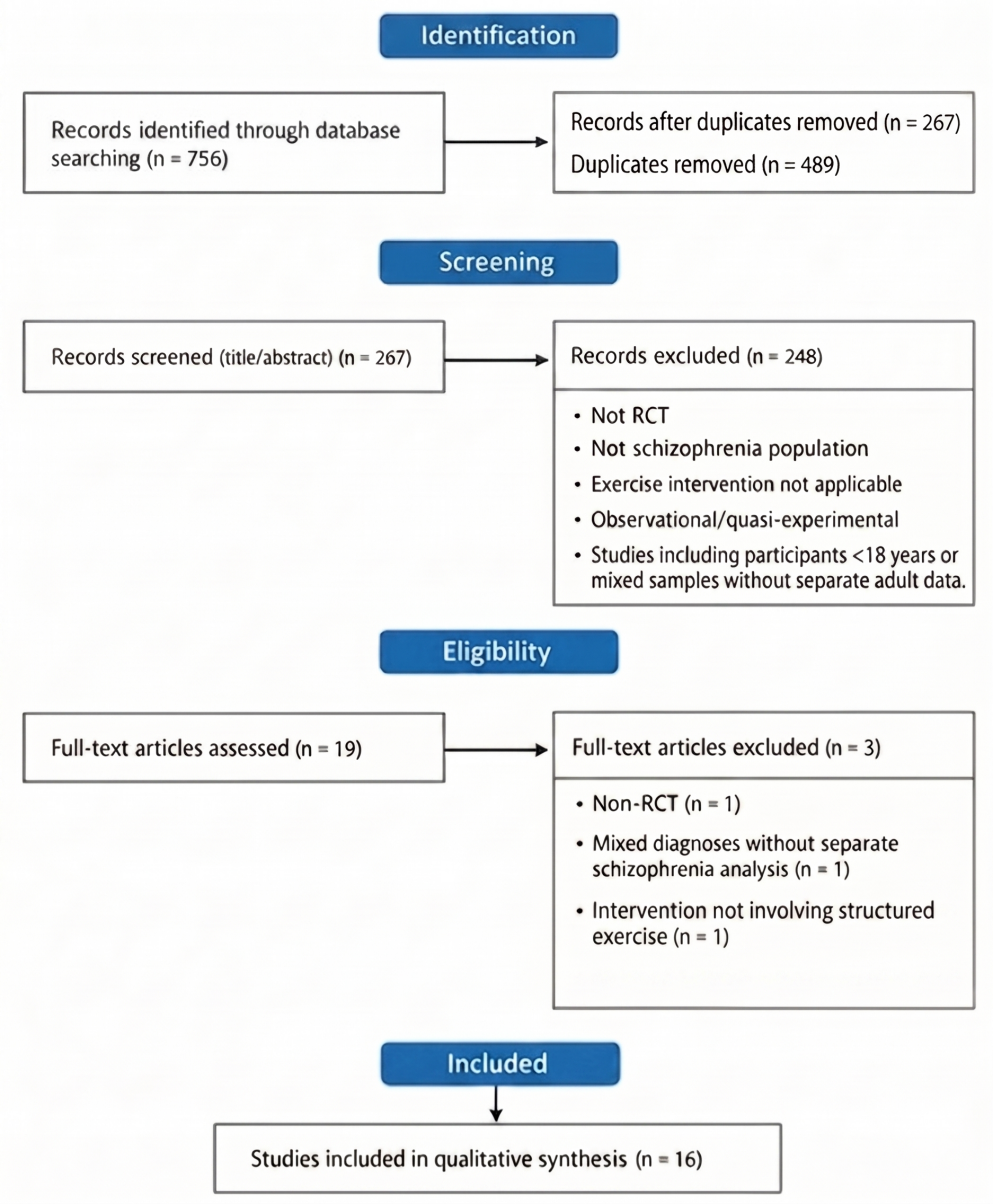
**Flowchart of the process adopted to identify, select, determine eligibility, and include studies in the systematic review**. RCT, randomized controlled trial. Source: Prepared by the authors.

For descriptive purposes, measures of central tendency and dispersion were calculated for selected study characteristics. The median (Md) and interquartile range (IQR) were used to summarize variables, including sample size, number of participants per group, and intervention duration. These descriptive statistics were used solely to characterize the included studies and not for inferential comparisons between trials. The final selection of studies is presented in the PRISMA flow diagram (Fig. 1).

## Results

The included studies were organized to provide a clear and integrated overview of the methodological, sampling, and clinical characteristics of the analyzed trials. Initially, a detailed characterization of the studies was carried out, including the country where the studies were conducted, type of physical exercise intervention, clinical context of the participants (outpatient, community, or hospital), duration of the programs, and number of participants per group. Table 1 (Ref. [8, 9, 10, 15, 16, 17, 18, 19, 20, 21, 22, 23, 24, 25, 26, 27]) summarizes this information, allowing a general overview of the samples and experimental protocols.

**Table 1.** **Characteristics of the RCTs included in the systematic review: author, clinical setting, country, intervention duration, and sample size (total and per group)**.

Author (year)	Clinical setting	Country	Intervention duration (weeks)	Sample size	Number of participants in the experimental group (EG)	Number of participants in the control group (CG)
Silva *et al*. [8]	outpatient	Brazil	20	34	21 (RESEX, n = 12 + CONCEX, n = 9)	13
Curcic *et al*. [9]	institutionalized	Serbia	12	80	40	40
Brobakken *et al*. [10]	outpatient	Norway	12	48	25	23
Maurus *et al*. [15]	outpatient and institutionalized	Germany	12	15	7	8
Ho *et al*. [16]	institutionalized	China	12	153	102 (Tai Chi + exercise)	51 (wait-list control)
Tous-Espelosin *et al*. [17]	outpatient	Spain	20	112	59	53
Andersen *et al*. [18]	outpatient	Norway	12	82	41	41
Bang-Kittilsen *et al*. [19]	outpatient	Norway	12	82	43	39
Kurebayashi *et al*. [20]	institutionalized	Japan	8	22	5	17
Wang *et al*. [21]	outpatient	Taiwan	12	62	33	29
Kimhy *et al*. [22]	outpatient	United States	12	33	16	17
Cheng *et al*. [23]	outpatient	Taiwan	8	54	26	28
Ryu *et al*. [24]	outpatient	South Korea	16	60	30	30
Lo *et al*. [25]	outpatient	China	12	51	17	18
Shimada *et al*. [26]	outpatient	Japan	12	31	16	15
Khonsari *et al*. [27]	outpatient	Iran	8	40	20	20

Note: EG, experimental group; CG, control group; RESEX, resistance exercise; CONCEX, concurrent exercise; outpatient, participants receiving outpatient care; institutionalized, inpatient or hospitalized participants; wait-list control, participants receiving standard care while awaiting intervention; usual care, standard psychiatric treatment without structured physical exercise; RCTs, randomized controlled trials.

Descriptive analyses of the methodological characteristics of the included studies revealed a remarkable consistency in the duration of the interventions, with a median of 12 weeks (IQR = 12), adopted in 10 (62.5%) of the sixteen trials. The total sample size varied widely, ranging from 15 to 153 participants (Md = 52; IQR = 47), reflecting the coexistence of pilot trials and larger studies. The experimental groups had a median of 25 participants (IQR = 24), while the control groups exhibited an equivalent median (Md = 25; IQR = 22), indicating a general balance between the conditions compared.

The mean number of participants per study was 59, evidencing moderate sample sizes in most trials. Taken together, these data demonstrate heterogeneity in sample size, but methodological uniformity regarding the duration of the interventions, which were mostly concentrated in short-term to medium-term physical exercise programs.

Table 2 (Ref. [8, 9, 10, 15, 16, 17, 18, 19, 20, 21, 22, 23, 24, 25, 26, 27]) describes the exposure variables related to the physical exercise programs: category of physical exercise or modality, weekly frequency (days), session duration (minutes), intensity (when reported), and type of treatment received by the control group (CG) or comparison with other modalities.

**Table 2.** **Characteristics of structured physical exercise interventions and control conditions in the included RCTs**.

Description of the exposure variable(s)					
Author (year)	EG				CG
	exercise modality	frequency (sessions/ week)	session duration (minutes)	intensity	treatment
Silva *et al*. [8]	RESEX (resistance) and CONCEX (concurrent training)	3	60	60–80% 1RM (endurance); moderate 60–70% HRmax (aerobic)	usual outpatient treatment (without exercise)
Curcic *et al*. [9]	prescribed physical activity program (aerobic exercise)	4	45	moderate (65–75% HRmax)	conventional treatment, hospitalized (inpatient) patients
Brobakken *et al*. [10]	AIT	2	30–40	high intensity interval training	2 initial AIT sessions + encouragement for autonomous exercise—without regular supervised training.
Maurus *et al*. [15]	resistance training	3	50	moderate-vigorous	body balance and toning program (active control)
Ho *et al*. [16]	Tai Chi/moderate physical exercise	3	45–60	moderate	wait-list control + standard institutional rehabilitation care (medication and usual treatment)
Tous-Espelosin *et al*. [17]	combined workout: low-volume HIIT + resistance circuit	3	30–45	low-volume HIIT (<10 min of high-intensity time per session) and resistance	usual outpatient treatment
Andersen *et al*. [18]	HIIT	2	30–40	high intensity (high effort intervals on the treadmill)	outpatients with schizophrenia in usual treatment; computer gaming skills training (active control for social/sedentary engagement)
Bang-Kittilsen *et al*. [19]	HIIT on a treadmill (EG) and active video games (Nintendo Wii Sports: bowling, golf, tennis (CG)	2	45	EG: 8 min warm-up; 4 × 4 min at 85–95% HRmax with 3 min active recovery at 70% HRmax; 5 min cool-down; CG: low to moderate (free body movements, no specific HR target)	from psychiatric outpatient follow-up
Kurebayashi *et al*. [20]	low-intensity physical exercise	3	30–45	light	typical treatment for psychiatric hospitalization (pharmacotherapy + inpatient care), without structured exercise
Wang *et al*. [21]	AE	3	30–45	moderate-vigorous	patients receiving antipsychotics, usual community treatment.
Kimhy *et al* [22]	aerobic exercise (active-play video games + equipment AE)	3	30–45	moderate-vigorous aerobic (measured by VO_2_peak )	TAU (outpatient psychiatric care, no exercise)
Cheng *et al*. [23]	aerobic dance program	3	30–60	moderate	without intervention, usual outpatient care
Ryu *et al*. [24]	outdoor cycling	1	90	moderate	weekly occupational therapy (daily living skills)—without structured physical exercise
Lo *et al*. [25]	EG: HIIT; EC: psychoeducation without structured exercise	3	30–45	high intensity (HIIT protocol)	psychoeducation and usual treatment
Shimada *et al*. [26]	aerobic exercise (individual + group)	2	30–45	moderate	patients seen in outpatient services and maintaining usual psychiatric treatment.
Khonsari *et al*. [27]	aerobic exercise (multi-session program)	3	30–45	self-managed	pharmacological treatment (no exercise)

Note: EG, experimental group; CG, control group; AE, aerobic exercise; AIT, aerobic interval training; HIIT, high-intensity interval training; HRmax, maximum heart rate; 1RM, one-repetition maximum; TAU, treatment as usual; active control, structured non-exercise intervention; passive control, waiting list or no intervention condition; RCTs, randomized controlled trials; VO_2_peak, peak oxygen consumption; HR, heart rate.

Physical exercise interventions showed great diversity in terms of the modalities, intensities, and training structures used in clinical trials. Traditional aerobic interventions predominated [8, 10, 21, 22, 23], although some studies employed specific modalities, such as resistance training [8, 15], Tai Chi [16], and mixed programs involving low-volume HIIT combined with resistance circuits [17].

Weekly training frequency ranged from one to four sessions, with a predominance of three sessions per week, while the duration of the sessions ranged from 30 to 90 minutes, with an approximate median of 40–45 minutes. Regarding intensity, most interventions focused on moderate to vigorous intensity efforts, frequently monitored by physiological parameters such as percentage of maximum heart rate (typically 60–80% maximum heart rate (HRmax) or equivalent measures).

Notably, some studies adopted very high intensity protocols, especially those based on HIIT or aerobic interval training (AIT) [10, 18, 19, 25], while others used light exercise, such as low-intensity protocols [20]. The control groups were heterogeneous, varying between usual treatment without exercise [8, 21, 23], waiting lists [16], active non-physical interventions (psychoeducation, cognitive training, active video games) [18, 19, 25], or alternative programs of unstructured physical activity [9, 24].

The synthesis of clinical outcomes assessed by randomized clinical trials reveals consistent effects of physical exercise on multiple domains of schizophrenia (Table 3, Ref. [8, 9, 10, 15, 16, 17, 18, 19, 20, 21, 22, 23, 24, 25, 26, 27]). Most studies that used standardized psychopathological scales demonstrated significant reductions in general symptoms and, especially, in negative symptoms, with aerobic and HIIT protocols standing out [8, 9, 16, 21, 24]. Trials that included measures of cardiorespiratory fitness (VO_2_max or peak oxygen consumption (VO_2_peak)) reported significant improvements in aerobic capacity, often greater than those observed in control groups [10, 17, 23, 25].

**Table 3.** **Outcome measures and main post-intervention findings of the included RCTs**.

Author (year)	Measures, instrument(s) and variables analyzed	Main clinical outcome(s) post-intervention
Silva *et al*. [8]	PANSS	Significant reduction in total PANSS symptoms and positive symptoms, and improvement in negative symptoms in RESEX.
	SF-36, “role-physical” domain	Improvement in the “role-physical” domain of the SF-36 in the endurance group.
	Muscle strength tests (1RM arm extension, bench press)	Increase in muscle strength (1RM) in upper limbs.
	Serum markers: BDNF, IGF-1, IGFBP-3	No significant changes in BDNF, IGF-1, and IGFBP-3.
Curcic *et al*. [9]	PANSS—positive and negative symptoms and general psychopathology	Significant increase in VO_2_max in the EG after 12 weeks vs. control.
	Cardiopulmonary exercise test (VO_2_max)	Significant improvement in global symptoms and in the general psychopathology subscale of the PANSS in the EG.
		Significant reduction in the total PANSS score in the EG compared to the CG.
		Indicative that prescribed physical activity can alleviate clinical symptoms of schizophrenia when added to usual treatment.
Brobakken *et al*. [10]	VO_2_peak	Significant improvement in VO_2_peak in the EG compared to the CG after 12 weeks.
	Cardiometabolic measurements: weight, BMI, waist circumference, blood pressure, lipids, and glucose	No significant differences between groups in weight, BMI, waist circumference, blood pressure, lipids, or glucose in the post-test.
		Weight gain and BMI in the CG group vs. stability in the TG group.
Maurus *et al*. [15]	WHO-DAS 2.0, GAF, general and physical functioning measures	No significant difference in WHO-DAS (primary outcome).
		Significant improvement in GAF over time, more marked in the resistance group.
		Safe and feasible intervention.
		Good adherence, with no relevant adverse events.
Ho *et al*. [16]	Standardized psychiatric interviews (psychiatric status scales for psychotic, negative, and depressive symptoms)	Both groups (Tai Chi and physical exercise) showed a reduction in motor deficits compared to the CG.
	Self-report questionnaires	Tai Chi: increased working memory (backward digit span) and increased average cortisol.
	Performance tasks (e.g., forward and reverse digit span for memory)	Exercise: reduction in negative and depressive symptoms, improved memory (forward digit span) and function in activities of daily living, in addition to an increase in average cortisol.
	Measures of motor coordination	Both intervention groups presented fewer symptoms than the CG, with the exercise group showing better symptom management than the Tai Chi group.
	Salivary cortisol (average)	
Tous-Espelosin *et al*. [17]	Maximum cardiopulmonary exercise test on a cycle ergometer (VO_2_peak); body composition measurements; biochemical markers.	Significant increase in cardiorespiratory fitness (VO_2_peak, ventilatory threshold, peak HR) in the exercise group.
		Maintenance of body composition; no significant changes in TAU.
Andersen *et al*. [18]	VO_2_max (measured directly on a treadmill)	No significant difference between HIIT and control in VO_2_max at the end of 12 weeks.
	ActiGraph accelerometer (physical activity level)	No significant change in overall physical activity level or body composition between the groups.
	Bioelectrical impedance (body composition: weight, BMI, fat)	Increased workload on the treadmill was observed in HIIT.
		Approximately 47% of HIIT participants had a ≥5% increase in VO_2_max (individual clinical response).
Bang-Kittilsen *et al*. [19]	PANSS (5-factor model); CDSS; VO_2_max (cardiopulmonary exercise test)	HIIT increased VO_2_max and reduced depressive symptoms (CDSS) and specific components of the PANSS compared to active video games.
		Effects maintained in a 4-month follow-up.
Kurebayashi *et al*. [20]	Scale of psychiatric symptoms	After statistical adjustments (negative symptoms + days of hospitalization), the light exercise group showed a significant improvement in overall neurocognition compared to the CG.
	Global neurocognitive assessment (neuropsychological battery)	The authors suggest that light exercise may accelerate recovery and allow for earlier discharge.
	Length of hospitalization (days)	
Wang *et al*. [21]	PANSS	Significant reduction in negative symptoms and general psychopathology in the AE group vs. CG.
		Improvement in positive symptoms during 12 weeks in the AE group.
		Greater reduction in overall condition severity.
		Effects partially maintained up to 3 months of follow-up (for negative symptoms).
Kimhy *et al* [22]	Cardiorespiratory fitness test (VO_2_peak, mL/kg/min)	Significant improvement in aerobic fitness in the exercise group (+18% in VO_2_peak) vs. small decline in TAU.
	MCCB–neurocognitive composite score	Improvement in overall neurocognitive function (MCCB: +15.1%) vs. worsening in the CG (-2.0%).
	Serum BDNF level	Increase in serum BDNF, and changes in VO_2_ and BDNF predicting part of the cognitive improvement.
Cheng *et al*. [23]	Body weight	Significant reduction in weight and BMI in the EG.
	BMI	Significant improvement in flexibility.
	Flexibility	Improved cardiorespiratory fitness at the end of 8 weeks.
	Muscular endurance	Improved muscle endurance in the immediate post-test.
	Cardiorespiratory endurance	Fitness effects maintained in the follow-up.
Ryu *et al*. [24]	BPRS	Significant improvement in psychotic symptoms (BPRS) in the cycling group vs. CG.
	BDI	Reduction in depression, state anxiety, and trait anxiety in the cycling group.
	STAI	Improvement in overall functioning (GAF) in the EG.
	RSES	Improvement in executive function (accuracies and categories in the WCST).
	GAF	Significant increase in objectively measured physical activity (pedometer—steps/day).
	WCST	No difference in the total K-PASE score, but there was a difference in objectively measured daily steps.
	Physical Activity Scale (K-PASE)	
	Pedometer (steps/day)	
Lo *et al*. [25]	MST for sleep-dependent procedural memory consolidation; measures of cognitive performance and sleep (described in more detail in the full article)	HIIT and AE improved sleep-dependent procedural memory (SDM) consolidation compared to psychoeducation, with a more robust effect for HIIT.
Shimada *et al*. [26]	Standardized measures of cognitive performance	Significant improvement in overall cognitive function in the TAU + AE group compared to TAU alone after 12 weeks.
	General cognitive function questionnaires	AE showed a trend toward improvement in domains such as attention, working memory, and processing speed (greater effects in the EG).
Khonsari *et al*. [27]	PANSS (5-factor model); CDSS; VO_2_max (cardiopulmonary exercise test)	HIIT increased VO_2_max and reduced depressive symptoms (CDSS) and specific components of the PANSS compared to active video games.
		Effects were maintained at the 4-month follow-up.

Note: PANSS, Positive and Negative Syndrome Scale; SF-36, Short Form Health Survey (36 items); 1RM, one-repetition maximum; BDNF, brain-derived neurotrophic factor; IGF-1, insulin-like growth factor 1; IGFBP-3, insulin-like growth factor binding protein 3; VO_2_peak/VO_2_max, peak/maximal oxygen consumption; peak HR, peak heart rate; MCCB, MATRICS Consensus Cognitive Battery; BPRS, Brief Psychiatric Rating Scale; BDI, Beck Depression Inventory; STAI, State-Trait Anxiety Inventory; RSES, Rosenberg Self-Esteem Scale; GAF, Global Assessment of Functioning; WCST, Wisconsin Card Sorting Test; K-PASE, Korean version of the Physical Activity Scale for the Elderly; CDSS, Calgary Depression Scale for Schizophrenia; MST, motor sequence task; WHO-DAS 2.0, World Health Organization Disability Assessment Schedule 2.0.; CG, control group; EG, experimental group; AE, aerobic exercise; HIIT, high-intensity interval training; RCTs, randomized controlled trials; BMI, body mass index; TAU, treatment as usual.

Furthermore, cognitive benefits were also recurrent, especially in executive functions, processing speed, working memory, and sleep-dependent memory consolidation, particularly in interventions involving aerobic exercise or HIIT [22, 24, 25, 26]. Studies that assessed functional markers and measures of global functioning (such as Global Assessment of Functioning (GAF) and World Health Organization Disability Assessment Schedule (WHO-DAS)) showed moderate improvements [15, 24], while interventions focused on metabolic management, body composition, or cardiometabolic parameters presented more heterogeneous results [10, 18].

To enhance the interpretation of intervention effects, the results were additionally analyzed according to the type of control condition. Studies comparing SPEs with passive controls (e.g., waiting list conditions) generally demonstrated larger effect sizes, particularly in negative symptoms, global functioning, and cardiorespiratory fitness [16]. In contrast, studies using TAU as the control condition showed consistent, although more moderate, improvements, reflecting the additive effect of exercise to standard psychiatric care [8, 21, 23].

When compared to active non-exercise interventions (e.g., psychoeducation, cognitive training, or active video games), the effects of exercise were more heterogeneous, with some studies demonstrating superiority of exercise, particularly for physical fitness and specific cognitive domains, while others showed comparable outcomes between conditions [18, 19, 25]. This pattern suggests that part of the observed benefits may be influenced by engagement in structured activities, although exercise appears to provide additional physiological and neurocognitive advantages.

The risk of bias assessment revealed moderate methodological variability across the included trials. Six studies were classified as low risk of bias, while ten presented some concerns according to the RoB 2 criteria. The domains of randomization process and outcome measurement were generally rated as low risk or with some concerns. However, potential bias related to deviations from intended interventions and lack of blinding was frequently identified, which is expected in exercise-based interventions. These limitations should be considered when interpreting the magnitude and consistency of the reported effects [13, 14].

## Discussion

The current study aimed to systematically synthesize the evidence from randomized clinical trials that investigated structured physical exercise programs in individuals with schizophrenia and the effects on psychiatric symptoms and other clinically relevant outcomes. Overall, the findings consistently indicate that structured physical exercise interventions can produce beneficial effects on core dimensions of the disorder, particularly negative symptoms, overall functioning, and cardiorespiratory fitness, with additional improvements observed in certain cognitive domains. Across the analyzed trials, reductions in PANSS and BPRS scores were frequently reported, supporting the hypothesis that physical exercise may influence neurobiological mechanisms associated with the pathophysiology of schizophrenia, as suggested by recent literature [17].

Stratification of control conditions contributed to a more nuanced interpretation of the findings. Studies employing passive controls (e.g., waiting lists) generally reported more pronounced improvements [16], whereas comparisons with TAU demonstrated consistent additional benefits of structured physical exercise, particularly for negative symptoms and physical fitness [8, 21, 23]. In contrast, when compared to active non-exercise interventions (e.g., psychoeducation or cognitive training), the effects were more heterogeneous [18, 19, 25], suggesting that engagement in structured activities may partially account for clinical improvements. However, physical exercise appears to confer additional neurobiological and physiological benefits, especially in cardiorespiratory fitness and specific cognitive domains [10, 17, 22, 24]. Overall, the type of control condition emerges as a key methodological factor in interpreting the effects of exercise interventions in schizophrenia.

Improvements in negative symptoms, as observed in studies such as those of Wang *et al*. [21], Kimhy *et al*. [22], and Curcic *et al*. [9] corroborate earlier meta-analytic evidence indicating that physical exercise is among the few non-pharmacological interventions capable of producing clinically meaningful effects in this domain. Reviews [2, 28] suggest that motivational and cognitive deficits may be partially responsive to interventions that enhance hippocampal connectivity, increase BDNF levels, and reduce inflammatory markers—mechanisms that were also reported in some of the trials included in this synthesis, such as by Kimhy *et al*. [22].

In the present review, longer-duration aerobic exercise interventions demonstrated positive effects not only on psychiatric symptoms but also on cardiorespiratory fitness, as evidenced by significant increases in VO_2_max or VO_2_peak in the studies by Brobakken *et al*. [10], Cheng *et al*. [23], and Tous-Espelosin *et al*. [17]. These findings are consistent with evidence reported by Suetani *et al*. [29] and Kimhy *et al*. [30], who identified reduced physical fitness as one of the strongest predictors of mortality and functional disability in individuals with schizophrenia. Therefore, by improving aerobic capacity, physical exercise simultaneously contributes to both psychiatric and medical outcomes.

Despite the predominance of beneficial effects, not all studies reported homogeneous responses. Andersen *et al*. [18], for example, observed no significant differences in mean VO_2_max between intervention and control groups, although nearly half of the participants allocated to HIIT achieved an individual clinically relevant response (≥5%). This interindividual variability, also described by Ross *et al*. [31], may reflect differences in disease stage, adherence rates, antipsychotic medication effects, comorbidities, or biological variability in responsiveness to exercise.

The heterogeneity observed across the included trials may be explained by several methodological and clinical factors. These include differences in exercise modalities (e.g., aerobic training, resistance training, HIIT, Tai Chi, and combined protocols), variations in intervention intensity and duration, distinct clinical settings (outpatient versus institutionalized populations), and heterogeneity in participant characteristics, such as illness duration, medication regimens, and baseline physical fitness. Additionally, the studies employed different outcome measures and assessment instruments, which may have contributed to variability in the magnitude and direction of the reported effects.

Combined exercise interventions, such as those integrating HIIT and resistance training, as reported by Tous-Espelosin *et al*. [17], also yielded promising results, particularly regarding improvements in physical fitness and maintenance of body composition. These findings align with the literature emphasizing that multicomponent exercise programs may potentiate synergistic effects through the integration of cardiovascular and muscular stimuli, thereby promoting greater neural and functional plasticity [32].

Another relevant aspect concerns the cognitive effects observed following exercise interventions. Studies by Kimhy *et al*. [22], Ryu *et al*. [24], and Lo *et al*. [25] reported improvements in executive functions, working memory, and sleep-dependent memory consolidation—domains that are consistently impaired in schizophrenia. These findings are supported by neurobiological evidence demonstrating that aerobic exercise can increase hippocampal volume, modulate frontoparietal networks, and promote BDNF-dependent neuroplasticity [33, 34].

Functional outcomes were also positively influenced by physical exercise interventions. Studies conducted by Strassnig *et al*. [35] and Maurus *et al*. [15] reported improvements in activities of daily living and global functioning, including increases in Global Assessment of Functioning (GAF) scores. These findings are particularly relevant, as global functioning represents a robust marker of autonomy and long-term prognosis in schizophrenia [36] and typically shows slower improvement than positive symptoms.

Importantly, low-intensity exercise programs, such as the intervention proposed by Kurebayashi *et al*. [20], also demonstrated beneficial effects, particularly on cognitive outcomes and length of hospital stay. These results suggest that even less vigorous exercise stimuli can yield clinically relevant benefits, in line with recent recommendations for psychiatric populations with limited exercise tolerance [37, 38].

Despite these positive results, methodological limitations were identified across the included trials, including substantial heterogeneity in the intervention protocols, variability in usual treatment across centers, lack of blinding in some studies, and heterogeneous outcome assessment instruments. These factors complicate direct comparisons between studies, as previously highlighted by Guo *et al*. [38], Shimada *et al*. [26], and Sisman *et al*. [39]. Despite these limitations, beneficial effects were consistently observed across different clinical contexts, including outpatient, inpatient, and community-based settings, reinforcing the overall robustness of the evidence.

Additionally, although most interventions lasted for between 8 and 12 weeks, sustained benefits were reported in the follow-up assessments in the studies by Wang *et al*. [21] and Bang-Kittilsen *et al*. [19]. This maintenance of effects suggests that physical exercise may function not only as an acute intervention but also as a longitudinal component of schizophrenia treatment, consistent with the integrated care models proposed by Suetani and Vancampfort [29].

The current review presents limitations inherent to both the topic and the available body of evidence. Chief among these is the substantial heterogeneity of exercise protocols, encompassing differences in modality, intensity, frequency, supervision, and duration, which precluded quantitative meta-analysis. Additional limitations include variability in control conditions and generally modest sample sizes, which restrict the generalizability of the findings.

Conversely, this review exhibits several methodological strengths. It exclusively included RCTs, the gold standard for intervention research, thereby enhancing internal validity. The literature search was comprehensive, prospectively structured, and conducted across multiple international databases over a ten-year period. Furthermore, the detailed characterization of exercise protocols and the integration of psychiatric, cognitive, and functional outcomes provide a more comprehensive clinical perspective than prior reviews, which often focused solely on symptom reduction.

## Conclusions

The current review indicates that interventions of varying intensities and modalities can produce relevant clinical benefits, suggesting that physical exercise offers a range of possibilities, adaptable to the needs, abilities, and preferences of each patient. This flexibility is particularly important in schizophrenia, considering the psychosocial, motivational, and functional barriers frequently associated with the disorder. Thus, the results presented here support the incorporation of physical exercise programs into mental health services, both in outpatient and institutional settings, as an accessible, low-cost, and potentially transformative care strategy for the clinical course of the disease.

Finally, although the findings support the therapeutic potential of physical exercise, there is still a need for greater standardization of protocols, methodological improvements, and studies that explore the mechanisms involved in the identified effects, with greater precision. Future studies should explore characteristics that modulate the response to exercise (intensity, volume, adherence, interactions with pharmacological treatment, disease stage) and evaluate long-term outcomes, including, for example, social reintegration, quality of life, and autonomy. Even so, based on the current evidence, physical exercise emerges as a robust and multidimensional non-pharmacological intervention, capable of significantly contributing to the advancement of more integrated, humane, and effective clinical practices focused on the care and treatment of schizophrenia.

## Availability of Data and Materials

All data generated or analyzed during this study are included in this published article.

## Supplementary Material

Supplementary material associated with this article can be found, in the online version, at https://doi.org/10.62641/aep.v54i3.2198.
